# Exploring the Effectiveness of Immersive Virtual Reality Rehabilitation for Parkinson’s Disease: A Narrative Review

**DOI:** 10.3390/jcm14196858

**Published:** 2025-09-28

**Authors:** Roxana Nartea, Daniela Poenaru, Mariana Isabela Constantinovici, Claudia Gabriela Potcovaru, Delia Cinteza

**Affiliations:** 1 Department of Physical and Rehabilitation Medicine, Carol Davila University of Medicine and Pharmacy, 050451 Bucharest, Romania; daniela.poenaru@umfcd.ro (D.P.); m.constantinovici@umfcd.ro (M.I.C.); claudia-gabriela.potcovaru@drd.umfcd.ro (C.G.P.); delia.cinteza@umfcd.ro (D.C.); 2 National Institute for Rehabilitation, Physical Medicine and Balneoclimatology, 030079 Bucharest, Romania

**Keywords:** immersive virtual rehabilitation, Parkinson’s disease, rehabilitation, virtual reality

## Abstract

Parkinson’s disease (PD) presents an association of motor and non-motor impairments that impact the independence and quality of life of individuals. Rehabilitation programs must address multiple domains, simultaneously maintaining patients’ adherence and the implications of the disease. Immersive virtual-reality-based rehabilitation (IVRBR) is a promising alternative tool, or can be used in conjunction with traditional or passive programs, using interactive tasks in valid environments with specific training programs adapted to each individual’s needs. This narrative review synthesizes the medical literature published in the last decade from PubMed, Scopus, and Web of Science, on the effectiveness, limitations, and implementations of IVRBR in PD patients. Evidence from RTCs and non-RTCs suggests that IVRBR can improve balance, motor learning, and dual task performance. At the same time, the evidence suggests that it can improve cognitive and emotional status. The integration of objective assessment tools (motion and posture analyses, wearable sensors, center of pressures and machine learning models capable of predicting freezing gait-FoG) enhances clinical and individualized rehabilitation programs. However, the evidence base remains limited, with a small sample size, heterogeneity in measured outcomes, and short follow-up duration. In general, reported adverse reactions were minor, but required standardized reporting patterns. Implementation is challenging due to the equipment cost and varying technological demands, but also due to patient selection and training of the medical personnel. IVRBR is a feasible and engaging alternative or can form part of an individualized rehabilitation program in PD patients; however, future large RTCs, long-term follow-up with standardized protocols, cost-effectiveness analyses, and integration of predictive modeling are essential for its broader clinical usage.

## 1. Introduction

One percent of the population over 60 years old has Parkinson’s disease (PD), one of the most prevalent slow-progressing neurodegenerative diseases in the world [1]. Its prevalence is rising with age. In addition to non-motor problems (including dementia, dysautonomia, and cognitive deficiencies), patients with PD also suffer from motor disorders, such as (1) resting tremor, (2) bradykinesia, (3) rigidity, and (4) postural instability, which impair balance, gait, and movement quality. Other associated features are loss of smell, sleep dysfunction, mood disorders, excess salivation, constipation, and excessive periodic limb movements during sleep [2,3].

Patients with Parkinson’s disease (PD) are up to six times more likely than healthy individuals to have cognitive impairment. Consequently, in addition to the main motor features, Parkinson’s disease (PD) is associated with numerous non-motor symptoms that considerably increase the overall burden of the illness [4,5]. Cognitive impairment and Parkinson’s disease (PD) impact many cognitive areas, including executive processes, memory, attention, and visuospatial skills [4]. As a result, gait abnormalities like slower speed and shorter steps, as well as poor postural control, appear to be commonly linked to cognitive impairment [6]. When combined, these characteristics cause a significant impairment in everyday activities and quality of life, placing a heavy load on families and caregivers even in the early stages of Parkinson’s disease [2,3,7,8]. In addition to conventional applications of physiotherapy and rehabilitation, the use of technological interventions is a promising feature of the complex PD rehabilitation framework, and, in particular, virtual reality approaches are suggested as potentially valuable tools for these patients [9].

The fundamental neural pathways supporting VR-based therapy are mirror neurons from the primary motor cortex, dorsal premotor cortex, and supplementary motor area [7,10]. According to data from human neuroimaging, virtual reality’s effects on human brain plasticity and motor rearrangement may activate mirror neurons in the cerebellum and cortical and subcortical regions linked to motor control, thus stimulating the body’s sensorimotor system [11,12,13,14].

Immersive VR-based rehabilitation programs (IVRBR) use interactive settings that imitate real-life circumstances to create a safe and controlled environment for motor and cognitive training [9,13]. These applications use repetitive, task-specific activities with real-time feedback to stimulate neuroplasticity, adaptability, and motor learning. Compared to traditional therapy, IVRBR systems can be adjusted to meet the individual needs and skills of patients with Parkinson’s disease, thereby increasing motivation and engagement [9].

In addition to its physical advantages, IVRBR improves the psychosocial component of Parkinson’s disease. By facilitating involvement in virtual group activities or environments that replicate social interactions, virtual reality’s immersive and interactive features may help alleviate feelings of loneliness [6,15]. Given that Parkinson’s disease (PD) frequently involves both physical deficits and cognitive and emotional difficulties, this all-encompassing approach to rehabilitation is very beneficial.

In recent years, there has been a notable expansion in research utilizing computer-assisted devices within interventions aimed at enhancing treatment efficacy and improving the accuracy of clinical diagnosis and progress [16,17].

Despite the promising evidence, there are still several remaining gaps in the medical literature. Variations in study designs, IVRBR platforms, intervention durations, and outcome measures make it difficult to draw straight conclusions. The long-term sustainability of IVRBR-induced balance gains and their effect on fall rates are also poorly understood.

IVRBR therapies in Parkinson’s disease (PD) build upon core neurorehabilitation concepts (neuronal plasticity, motor learning, sensory–motor integration, and cognitive engagement), but recent clinical trials help us understand *how* these translate into functional improvements.

A comprehensive, non-systematic literature search was conducted using PubMed, Scopus, and Web of Science to identify relevant studies focusing on IVRBR and Parkinson’s disease. Keywords combined Parkinson disease with “virtual reality”, “immersive VR”, games-based rehabilitation”, “cognitive training”, “virtual games”, and “augmented reality”. Boolean operators (AND, OR) and abbreviations were utilized to strengthen and refine search results. The study limited itself to English-language papers published between 2015 and 2025 in order to discover the latest evidence. References from the selected articles were manually examined in order to find further relevant information.

This review aims to synthesize evidence with emphasis on intervention characteristics, outcomes, feasibility, and gaps in knowledge. Risk of bias in RCTs was considered narratively based on reported methods, but no formal meta-analysis was performed, mainly due to the heterogeneity of designs and methods.

## 2. Theoretical Foundations of Immersive Virtual-Reality-Based Rehabilitation in Parkinson’s Disease

Virtual-reality-based rehabilitation (VRBR) in Parkinson’s disease (PD) builds upon core neurorehabilitation concepts, including neuronal plasticity, motor learning, sensory-motor integration, and cognitive engagement. Recent clinical trials help us understand how to translate these into functional improvements.

### 2.1. Inducing Neuroplasticity and Motor Recovery

Despite dopaminergic neurodegeneration in the basal ganglia, the brain maintains the capacity for reorganization. Evidence from RCTs shows that VRBR interventions, especially when combined with motor imagery (MI) or conventional physical therapy (PT), produce significant improvements in motor symptoms. For example, a trial of PD patients receiving VRBR + MI + routine PT for 12 weeks showed reductions in resting tremor, rigidity, postural instability, and gait impairment compared to PT alone [18].

Mechanistically, it seems that repetitive, task-oriented activities with feedback and a graded level of difficulty increase synaptic plasticity in the areas of the motor and premotor cortex, which in fact increases motor output. Also, home-based exergaming with full-body movements plus motion sensing has been shown to lead to measurable changes in resting-state functional connectivity and brain volume in PD patients with gait/balance disorders [19].

### 2.2. Sensory–Motor Integration and Balance Control

Participation and adherence are improved by immersive, game-like characteristics, which also improve quality of life (QoL) and activities of daily living (ADL). While IVRBR-based physiotherapy has been reported to provide more permanent gains in functional mobility than a traditional rehabilitation program alone, an umbrella review of meta-analyses (2023) indicated moderate benefits on ADL and QoL [20,21,22].

Additionally, immersive exergaming combined with intensive physiotherapy was shown to produce more sustained gains in functional mobility, especially under dual-task conditions, compared to physiotherapy alone. This suggests that the enriched environment afforded by VR can lead to better long-term maintenance of therapeutic effects [23].

**Putting these pieces together, a clinically optimal IVRBR program for PD should include the following:**

- Repetitive, task-oriented movements to engage neuroplasticity.
- Sensory challenges or manipulations (visual, proprioceptive, vestibular) to foster sensory–motor integration.
- Feedback (visual, auditory, perhaps haptic) to enable error correction and motor learning.
- Cognitive or dual-task components to promote executive control and attention, especially for real-world tasks.
- Engaging, motivating formats to promote adherence, long duration, and thus durable gains.

## 3. Evidence Synthesis of Immersive Virtual Reality in Parkinson’s Disease Rehabilitation

**Figure 1:**
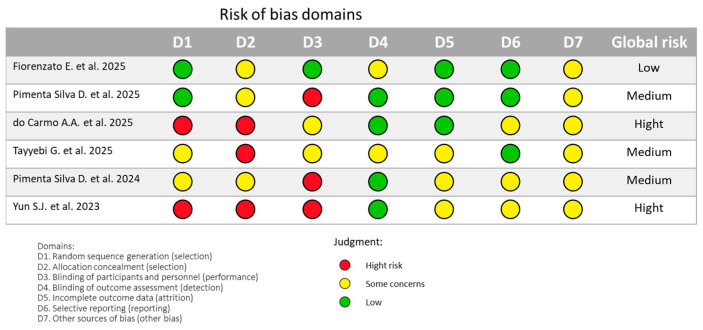
Risk of bias for RCTs [21,23,24,28,29,33].

The current literature on immersive virtual-reality-based rehabilitation (IVRBR) for Parkinson’s disease (PD) rehabilitation demonstrates promising therapeutic potential but remains limited by methodological heterogeneity and small sample sizes. Of the 21 studies included in this review, only 6 were randomized controlled trials (RCTs), while the remainder consisted of quasi-experimental or observational designs, limiting causal inference (Supplementary Table S1) [21,23,24,25,26,27,28,29,30,31,32,33,34,35,36,37,38,39,40]. The Cochrane Risk of Bias 2 (RoB2) tool was used to reveal recurrent limitations, including inadequate reporting of randomization procedures, allocation concealment, and the practical impossibility of blinding participants or therapists in RCTs studies using IVRBR interventions (see Figure 1).

Most studies enrolled fewer than 60 participants, with follow-up durations rarely exceeding two months, and used a wide range of outcome measures—including Timed Up and Go (TUG), Berg Balance Scale (BBS), 6-Minute Walk Test (6 MWT), PDQ-39, and HADS—complicating comparisons and synthesis.

### 3.1. Quantifiable Clinical and Functional Outcomes

Similarly to a typical physical therapy program without IVR, MindPodDolpdin enhanced mobility, motor ability, balance, and cognition after 6–12 weeks of use. One benefit of IVRBR was that the benefits persisted for 12 weeks following the therapy session [23].

A quasi-randomized study found that using music with IVRBR enhances executive function. The signal remains positive in the cognitive domain despite the study design’s limitations on assurance [31]. According to Carmo, virtual immersion enhances cognitive function and anxiety levels. They came to this conclusion after analyzing the outcomes from non-immersive and immersive training in Parkinson’s disease patients [24].

IVR for cognitive–behavioral treatment has been demonstrated to reduce depression and anxiety while increasing quality of life significantly. According to Tayyebi and associates, this improvement is noticed sooner in the emotional/quality of life sector than in the motor one [28].

IVRBR-assisted cognitive–motor dual-task training improves dual-task/executive task performance and balance. However, because it is not a randomized controlled comparison research, it provides limited evidence on the efficacy of this treatment [26].

HIIT aerobic training in IVRBR (cycling/rowing) increases TUG (*p* = 0.028), Tinetti (*p* = 0.046), PDQ-39 (*p* = 0.035), and MDS-UPDRS (*p* = 0.001) [30]. However, this data appears in a non-randomized trial, and while it may offer a positive signal for function/balance/QoL, it implies an increased probability of bias [30].

Virtual rowing and cycling, as well as exergames and walking, are activities performed in an immersive virtual environment that improve quality of life, executive functions, and balance; however, without well-chosen control groups, they remain only signals for future randomized studies that may provide more viable results [25,32,38,41,42].

Oña et al. found that the IVRBR-BBT can assess upper limb manual dexterity in patients with early Parkinson’s disease. This technological innovation has resulted in the development of the virtual Box and Block Test (IVRBR-BBT), a reliable tool for assessing manual dexterity [37].

From the point of view of balance, several meta-analyses confirm the benefit of using IVRBR. Kwon in 2023 (14 RCTs, *n* = 524) showed significant increases in BBS/ABC, but not in other motor indices and QoL [15]. A dose–response meta-analysis published in *Neurology* in 2025 highlights the significant BBS effect (WMD ≈ 3.6 points) and recommends performing sessions of ≤20 min duration, for 4–7 times per week, for a total of >40 sessions [43]. The overall favorable effect on balance is also demonstrated by De Natale et al. in their analysis from 2025 [4]. Apart from the previous ones, our analysis, based on newer studies, highlights the fact that the effect of IVRBR is more apparent when the study protocol includes tasks oriented on weight transfer/step strategy.

The primary target of IVRBR is balance/postural control, and thus IVRBR can be routinely used as an adjunct to individualized medical rehabilitation programs for patients with Parkinson’s disease [15]. The suggested protocol (for BBS) is ≤20 min/session, 4–7/week, 4–7 weeks, total >40 sessions; weight transfer tasks, stepping, postural response anticipation, with immediate feedback. Walking/6 MWT: use slightly longer sessions (≈21–40 min) in 4–7 wk windows, with dual-task and ecological scenarios (doors, hallways, obstacles) [44].

Examining gait, our analysis yields mixed results, with some improvements in gait speed, but not consistently on the 6 MWT/DGI [35,39]. The same conclusions were also highlighted by Kwon 2023, who found no robust differences in gait/ADL/UPDRS II–III, except for 10 MWT in some analyses [15].

### 3.2. Safety and Adverse Events

Safety profiles across studies suggest that IVRBR interventions are generally well tolerated, but careful monitoring is essential. In 2024, Silva and colleagues documented 144 adverse events (over a 12-week period −8.4% of sessions), the most of which were minor and included soreness, discomfort, and motor irregularities. Two of those incidents were serious, one of which resulted in withdrawal. The immersive “sense of presence” was directly associated with five falls, highlighting once again the significance of selection of proper game and monitoring during all rehabilitation programs [29]. Yun et al. (2023) and Honzíková et al. (2025) also confirm the viability of IVRBR if it is combined with safety protocols, by observing a few side effects, mostly mild cybersickness, and no significant injuries in their studies [27,33].

### 3.3. Cost-Effectiveness Considerations

There are few official economic analyses. According to studies conducted by Cancela-Carral et al. (2024) and Campo-Prieto et al. (2024), IVRBR has the ability to provide intense, captivating therapy with less in-person monitoring [26,44]. According to them, this aspect could reduce long-term healthcare expenses [26,44]. However, the need for therapist training, expensive initial equipment costs, and continuous maintenance remain to be obstacles to more widespread implementation. Future large-scale trials should incorporate cost-effectiveness assessments to help guide clinical and administrative decisions.

### 3.4. Implementation Barriers

The practical implementation of IVRBR may be limited by a number of aspects:

- Participation may be limited by patient-specific circumstances, such as advanced disease stage, cognitive impairment, or vulnerability to cybersickness.
- Standardization of protocols is further complicated by technological heterogeneity, such as head-mounted displays versus commercial consoles and immersive versus non-immersive systems.
- Because the majority of the experiments were carried out in extremely controlled settings, concerns regarding workflow integration and scalability were raised.
- Clinical training needs and alignment with current rehabilitation procedures are still not well understood.

Furthermore, best practice consensus and comparability are negatively impacted by differences in task complexity, session time frame, and outcome measures.

## 4. Integration of Objective Assessment Tools in VR Rehabilitation for Parkinson’s Disease

Systematic reviews and meta-analyses published over the past decade have demonstrated that virtual reality improves rehabilitation outcomes in patients with PD compared with conventional or passive rehabilitation programs [15,40,45,46,47,48]. With minimal impact on gait speed, motor function, and day-to-day activities, virtual reality training may improve balance (as assessed by the Berg Scale, postural stability), stride length, and coordination [15,47,48]. Utilizing head-mounted displays (HMDs) that provide multimodal stimulation, high presence, and repetitive training potential, immersive virtual reality (VR) could be an important part of rehabilitation programs [42]. A comprehensive, objective, sensory, and functional-based assessment, able to detect even small, temporary fluctuations, is the vital component to maximize the benefits of medical rehabilitation programs in patients with Parkinson’s disease.

Reaction time tests, motion capture, body-worn sensors, physiological sensors, depth-/camera-based tracking, and digital biomarkers caused by movement or cognitive tasks are examples of objective assessment methods [25,28,49,50]. The utility of objective measurements in immersive VR situations for PD is demonstrated by recent studies.

- Assessment embedded in VR tasks: the reaction time wall test is part of the VR environment and is used not only as a measurement but also as a training tool, a concept known as ‘dual use’ [25]. Similarly, finger tapping tasks in immersive VR allow direct measurement of rhythmicity metrics (frequency, variability) that correlate with PD motor impairment [49].
- Comprehensive assessment with multimodal sensing: the integration of VR HMD-based environments with force platforms (for CoP), physiological sensors (heart rate, stress/arousal), and wearable sensors (glove sensors), in addition to clinical scales, ensures a thorough and multi-dimensional measurement of motor, balance, and cognitive function [26,50].
- VR’s role in remote monitoring: wearable sensors embedded in gloves, visualized via VR avatar or visualization dashboards, play a crucial role in remote monitoring [42,50]. They allow clinicians to observe the exact movement trajectories (in space/time), a significant advancement in telemedicine for PD rehabilitation.
- Safety and tolerance measurement as part of assessment: simulator sickness scores, CoP sway, stress/arousal before and after immersive exposure are objective or quasi-objective measures relevant to rehabilitation [38,42,50]. Both ensure the tolerability and quality of the intervention.
- Correlation with gold standard clinical tools: objective metrics obtained via IVR tasks often are compared with established clinical assessments such as the Timed Up and Go (TUG) test, the Mini-Mental State Examination (MMSE), and the Unified Parkinson’s Disease Rating Scale (UPDRS) to establish the validity, sensitivity, and relationship with fall risk or disease severity [28,42,50].

## 5. Freezing of Gait (FoG) and the Role of Predictive Modeling in Immersive VR Rehabilitation for Parkinson’s Disease

Freezing of gait (FoG) is among the most disabling manifestations of Parkinson’s disease (PD). It involves abrupt, short-lived episodes in which patients suddenly find themselves unable to begin or maintain walking, even though they intend to do so [51]. These unpredictable events increase their risk of falling, compromise their independent mobility, and reduce their quality of life. Because of its episodic and context-specific nature, FoG remains challenging to both monitor and treat effectively.

Recent progress in machine learning (ML) has opened up new opportunities to anticipate and manage FoG. It has been shown that computer algorithms trained on motion data can detect freezing episode precursors before they happen [52]. Dopaminergic drug level is a key factor, according to a recent study publish by Borzi and colleagues in 2021 [52]. The researchers demonstrated that gait parameters (determined by analyzing spatiotemporal gait metrics from wearable sensors) fluctuate significantly between the ON- and OFF-medication stages. This, in turn, affects the accuracy of FoG prediction [52]. The significance of including medication effects into assessment and intervention planning has been shown by this observation.

Immersive virtual-reality-based rehabilitation (IVRBR) platforms provide an ideal setting to merge these predictive tools with therapy. Such systems can recreate everyday environments where freezing is commonly triggered—for example, narrow corridors or crowded walkways [53]. Embedding ML models into these virtual contexts allows the platform to monitor gait in real time and adapt tasks based on the likelihood of an impending FoG episode [38,50]. This dynamic adaptation means that patients receive tailored support when they are most vulnerable, while still being challenged under safer conditions [28].

Medication timing further enriches the personalization of IVRBR. Patients may be required to perform more difficult exercises during ON-medication times in order to increase resistance and motor learning. With an emphasis on safety and gradual engagement, the same technology can automatically reduce work complexity during OFF-medication phases. This adaptability minimizes potential risks while ensuring that treatment continues to be effective against a range of motor state fluctuations [28,52].

Therefore, including FoG prediction into immersive VR rehabilitation has two advantages: it produces an interactive, engaging therapeutic environment and provides interventions that are responsive to each person’s physiological state in real time. This personalized, adaptive approach holds promise for reducing falls, improving mobility, and ultimately enhancing the quality of life of people living with Parkinson’s disease.

## 6. Discussion

The growing role of immersive virtual-reality-based rehabilitation (IVRBR) in Parkinson’s disease (PD) is highlighted in this review. Its ability to improve balance and postural control is supported by evidence, although its benefits on gait, ADLs, and cognition remain less clear. Additionally, the combination of predictive modeling and objective assessment techniques indicates that IVRBR may evolve from a training modality into a customized, adaptable therapy platform [21,23,27].

The greatest consistent advantage is in balance training. According to meta-analyses, IVRBR raises Berg Balance Scale (BBS) scores, particularly when programs are frequent, limited in length, and last longer than 40 sessions. On the other hand, gains in ADL, endurance, and gait speed are still uncertain. This most likely illustrates how PD gait deficits are multifaceted. Since they target both the motor and cognitive factors that contribute to mobility impairments, interventions that mimic real-world difficulties—like obstacle navigation or dual-task activities—seem to have the most possible potential [25,26,32].

The psychosocial results are also positive. Improved attention, mood, and quality of life have been linked to VR tasks that include social interaction, music, or executive function problems. However, additional validation is required, as these findings are primarily based on short, non-randomized experiments.

The incorporation of objective, multimodal assessment tools is one of IVRBR’s distinguishing advantages. Continuous, sensitive data is provided via measures including physiological stress indices, wearable sensor-derived gait metrics, and center of pressure sway. By supporting both monitoring and rehabilitation, these “dual-purpose” exercises can produce digital biomarkers that are ecologically valid, so facilitating remote care and adaptive therapy.

The use of machine learning (ML) for freezing of gait (FoG) is especially intriguing. ML models can predict FoG episodes and modify VR activities in real time by examining spatiotemporal gait parameters. Closed-loop therapy that is adapted to the patient’s physiological state is made possible by taking medication state (ON vs. OFF) into account, which improves prediction accuracy and safety.

The safety information is comforting, and the negative effects (such as muscle tiredness and cybersickness) are usually minor. Furthermore, IVRBR’s captivating and immersive qualities improve adherence and motivation, which may have long-term effects beyond treatment. Careful patient selection is still vital because tolerance varies depending on the severity of the disease and cognitive state.

Implementation obstacles include upfront expenses, physician training requirements, and the variety of devices and protocols. Strong health economic assessments are missing, despite preliminary analyses pointing to possible cost savings through tele-rehabilitation and reduced falls.

Small sample sizes, brief follow-up periods, inconsistent results, and a dearth of randomized controlled trials continue to limit the research. Longer follow-up, multi-center studies with sufficient power and standardized techniques are required. Cost-effectiveness should be a key endpoint in future studies, along with assessments of home-based programs and validation of predictive analytics.

## 7. Challenges and Future Directions for IVRBR in PD

A potential tool for Parkinson’s disease (PD) rehabilitation, immersive virtual reality (IVRBR) has the potential to improve motor function, balance, and cognitive engagement. However, several knowledge-based and practical obstacles exist in implementing IVRBR programs in routine clinical practice after they have been studied.

Infrastructure and technical barriers are two major challenges. Not all healthcare facilities are able to afford the substantial hardware, software, and also the continuing maintenance costs associated with establishing IVRBR systems. The implementation may also be compromised by technical problems and a lack of familiarity with the technology to medical personnel and patients.

User experience and accessibility also pose challenges. Patients with Parkinson’s disease may experience some discomfort during IVRBR sessions, for example, dizziness, cybersickness, or anxiety, especially if the virtual environment is not adjusted to their functional and motor levels. IVRBR interfaces must be patient-centered, adaptable, and easy to use in order to maximize patient participation. To optimize involvement, IVRBR interfaces must be user-friendly, flexible, and patient-centered. In the same manner, clinical integration demands ongoing support and professional education of the medical personnel on how to safely and successfully apply IVR therapies.

Despite the gamified and immersive aspects of IVRBR, it is still difficult to maintain patient adherence and motivation throughout prolonged therapy periods. As important as maintaining adherence to legal and moral requirements, such as privacy and data protection, are safety factors, such as fall risk and appropriate supervision.

In order to overcome these obstacles and maximize therapeutic impact, future research objectives will focus on these topics. It is essential to standardize intervention techniques, including session length, frequency, and intensity, in order to obtain some reliable outcomes. Sustained effects will be confirmed by examining the effectiveness and longevity of improvements in motor and cognitive function. Patient-specific outcomes may be improved by integrating VR into multidisciplinary care models and tailoring therapies according to illness stage and functional limitations. The first priority should continue to be given to ensuring inclusivity and accessibility, including for patients with physical or cognitive impairments.

Wider acceptance will be guided by assessments of practicality, cost-effectiveness, and resource allocation. Patient experience will be maximized through ongoing safety monitoring and the application of new technologies.

The safe, efficient, and fair integration of immersive learning will be made possible by addressing these issues through collaborative research and advancements in medical technology.

The safe, efficient, and equitable integration of IVRBR into PD rehabilitation will be made possible by addressing these issues through comprehensive research and clinical innovation. This will ultimately support individualized and long-term therapy that enhances patient functionality, promotes active participation, and improves quality of life.

## 8. Conclusions

**Figure 2:**
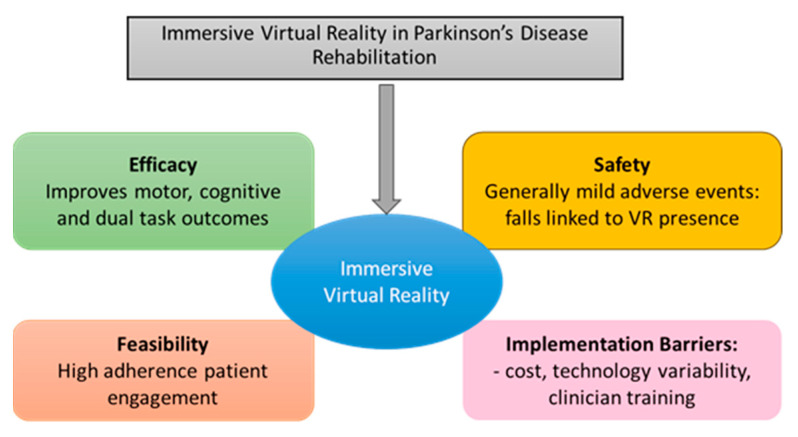
Strengthened IVRBR.

Immersive virtual reality is a feasible, safe, and engaging intervention for PD rehabilitation, with evidence supporting improvements in motor performance, cognitive function, dual-task ability, and quality of life. Both RCTs and feasibility studies confirm high adherence and generally mild adverse events, although careful supervision and game selection remain critical to minimize the risk. Implementation barriers—including cost, protocol variability, and patient-specific limitations—must be addressed for broader clinical integration (see Figure 2).

Strengthened by the available evidence, the following conclusions can be drawn:

- Efficacy: IVRBR is effective as an adjunctive therapy for improving motor, cognitive, and dual-task outcomes. Though effect sizes and long-term sustainability require confirmation in larger RTCs.
- Safety: adverse events are generally mild, with falls linked to IVRBR presence being the primary concern. Safety protocols are mandatory.
- Feasibility: high adherence and patient engagement support real-world application, particularly when interventions are tailored to individual functional and cognitive status.
- Implementation: cost, technology variability, and clinician training are key barriers; addressing these is essential for the implementation of IVRBR into routine medical practice.
- Future Directions: larger, standardized RCTs, long-term follow-up, integration of objective assessment tools (e.g., FoG prediction, real-time gait analysis) are needed. Also, consideration of medication timing is needed to optimize personalization and effectiveness.

Overall, IVRBR offers a clinically promising, patient-centered approach to PD rehabilitation that can complement traditional therapies. Further research is required to establish standardized protocols, confirm long-term benefits, and facilitate routine medical practice.

## Data Availability

Not applicable.

## Supplementary Materials

The following supporting information can be downloaded at https://www.mdpi.com/article/10.3390/jcm14196858/s1.

## Abbreviations

The following abbreviations are used in this manuscript:

6 MWT	6 Minute Walk Test
AE	Adverse effects
BBS	Berg Balance Scale (BBS)
CET	Concurrent exergaming training
CoP	Center of pressure
FoG	Freezing of gait
HADS	Hospital Anxiety and Depression Scale
HMDs	Helmet-mounted displays for safety and adverse events
IVR	Immersive virtual reality
IVRBR	Immersive virtual reality-based rehabilitation
MI	Motor Imagery
MOCA	Montreal Cognitive Assessment
PD	Parkinson disease
PDQ-39	Parkinson’s Disease Questionnaire-39
SEQ	Sequential training
SSQ	Simulator Sickness Questionnaire
TUG	Timed Up and Go
UPDRS	Unified Parkinson’s Disease Rating Scale
VR	Virtual reality
