# Supplementary material for: Exploring the Effectiveness of Immersive Virtual Reality Rehabilitation for Parkinson’s Disease: A Narrative Review
Source: J Clin Med. 2025 Sep 28;14(19):6858. doi: 10.3390/jcm14196858 (PMC12525201; doi:10.3390/jcm14196858)
Supplement: Supplementary file 1 [file jcm-14-06858-s001.zip › jcm-3866016-supplementary.pdf]

Supplementary Table 1

	Main author	Year	Study type	No. of patients	Inclusion criteria	Objective	Results	Limitations	VR/HMD type	Intervention duration	Primary outcomes
1	Pimenta Silva et al. [23]	2025	RCT	≈40 PD	PD Hoehn&Yahr II–III, clinically stable	Effectiveness of adding iVR to integrated rehab	Motor and balance benefits	Small N, short follow-up	HMD Oculus Rift	12 weeks, 5 sessions/day	UPDRS III, BBS, TUG
2	Fiorenzato et al. [21]	2025	Double-blind RCT	≈30	PD with MCI	Effect of iVR cognitive training on EF	Improvements in EF, memory	Small N, 4-week duration	HMD HTC Vive	4 weeks, 3 sessions/week	MoCA, EF battery
3	do Carmo et al. [24]	2025	Comparative (iVR vs non-iVR)	≈24–60	Stable PD	Impact on anxiety and cognition	iVR > non-iVR for anxiety	Non-randomized, small N	HMD Oculus Go	4 weeks	HADS, MoCA
4	Campo-Prieto et al. [25]	2024	Pre-post	≈20	PD ≤ Hoehn&Yahr III	Functional effects of iVR	Mobility improvements	No control group	HMD Oculus Quest	8 weeks	SPPB, TUG
5	Honzíková et al. [27]	2025	Pilot	≈28	PD II–III	Cognitive-motor dual-task with iVR	Dual-task improvements	No comparator	HMD HTC Vive	6 weeks	TUG dual-task, MoCA
6	Tayyebi et al. [28]	2025	RCT	≈45	PD with affective symptoms	iVR-CBT group effectiveness	Anxiety reduction, depression	Self-report, short follow-up	HMD Oculus Quest	8 weeks	HADS, PDQ-39
7	Pimenta Silva et al. [29]	2024	RCT (safety)	30	PD in intensive care	iVR safety in PD	Minor AEs, well tolerated	No clinical outcomes	HMD Oculus Rift	12 weeks	AE report
8	Cancela-Carral et al. [44]	2024	Pilot IntegraPark	16	PD moderate	HIIT + iVR feasibility	Good adherence	Small N	HMD Oculus Rift	3 months	VO2, UPDRS
9	Rosenfeldt et al. [30]	2024	Observational	25	PD and controls	iVR shopping task for IADL	Detects IADL deficits	Not interventional	HMD Oculus Quest	1 session	Performance shopping task
10	Rodríguez-Fuentes et al. [31]	2024	Quasi-RCT cluster	52	PD association members	ReViPark HIIT + iVR	Motor/QoL improvements	Cluster, confounders	HMD Oculus Rift	3 months	UPDRS, PDQ-39
11	Impellizzeri et al. [32]	2024	Quasi-RCT	40–60	PD without dementia	NMT + iVR on EF	EF benefits	Quasi-randomized	HMD HTC Vive	8 weeks	EF tests
12	Yun et al. [33]	2023	Feasibility	20–24	PD mild-moderate	Dual-task exergames	Good adherence, minor AEs	No control	HMD Oculus Quest	4 weeks	TUG dual-task
13	Campo-Prieto et al. [26]	2023	RT test validation	30–40	PD community	iVR reaction time vs fall risk	Significant correlation	Cross-sectional	HMD Oculus Rift	1 session	RT iVR, BBS

14	Besharat et al. [34]	2022	Lab	20–30	PD ± FoG	iVR environment doors/corridors	iVR influences gait kinematics	Not a therapeutic trial	HMD HTC Vive	1 session	Kinematics, FoG episodes
15	Goh et al. [35]	2021	Feasibility	12–20	PD with FoG	VR self-modeling video	Good acceptability	Small N	HMD Oculus Rift	4 weeks	FoG questionnaire
16	Lheureux et al. [36]	2020	Crossover	15–25	PD able to walk on treadmill	iVR gait autocorrelations	Normalization of gait pattern	Laboratory	HMD Oculus Rift	1 session	Stride autocorrelation
17	Oña et al. [37]	2020	Box&Blocks test validation	20–30	PD upper limb deficit	iVR Box&Blocks version validation	Good validity	Not interventional	HMD HTC Vive	1 session	Box&Blocks iVR
18	Sánchez-Herrera et al. [38]	2020	Mixed intervention	15–25	PD without dementia	iVR + serious games upper limb	Functional improvements	No RCT	HMD Oculus Rift	8 weeks	Motor tests
19	Cikajlo et al. [39]	2019	Parallel study	28–40	PD mild-moderate	3D VR training	Moderate benefits	Non-randomized	HMD Oculus Rift	6 weeks	UPDRS, balance tests
20	Kim et al. [40]	2017	Safety	20–30	PD + elderly	iVR gait safety	Minor AEs	Not interventional	HMD Oculus Rift	1 session	AE report
21	Bank et al. [42]	2018	AR feasibility	20–30	PD + stroke	AR games upper limb	Feasibility	AR, not iVR	AR glasses	—	Motor assessment

Abbreviation: 6MWT- 6 Minute Walk Test; AE -adverse effects; BBS - The Berg Balance Scale (BBS); CET - Concurrent exergaming training; HADS - Hospital Anxiety and Depression Scale; HMDs - Helmet-mounted displays for safety and adverse events; IVR- immersive virtual reality; IVRBR- immersive virtual reality-based rehabilitation; MOCA - Montreal Cognitive Assessment; PD – Parkinson Disease; PDQ-39 - The Parkinson's Disease Questionnaire; SEQ - Sequential training; SSQ - Simulator Sickness Questionnaire; TUG - Timed Up and Go; UPDRS- Unified Parkinson's Disease Rating Scale; VR-virtual reality
